# Effects of repeated cryostimulation exposures on sleep quality in swimmers during an intense training period

**DOI:** 10.1113/EP092293

**Published:** 2025-06-24

**Authors:** Coralie Arc‐Chagnaud, Benoit Dugué, Robin Pla, Romain Bouzigon, Laurent Bosquet, D‐Day Consortium, Olivier Dupuy

**Affiliations:** ^1^ Laboratoire MOVE – UR 20296 – Université de Poitiers Poitiers France; ^2^ Fédération Française de Natation Clichy France; ^3^ UFR STAPS Besançon, Laboratoire C3S (EA4660), Axe Sport Performance Université de Franche‐Comté Besançon France; ^4^ Inside the Athletes 3.0, Sport Performance Optimization Complex (COPS25) Besançon France; ^5^ Aurore concept Noisiel France; ^6^ École de Kinésiologie et des Sciences de l'Activité Physique (EKSAP), Faculté de Médecine Université de Montréal Quebec Canada

**Keywords:** cryostimulation, heart rate variability, sleep, swimmers

## Abstract

The aim of this study was to evaluate the effects of daily partial body cryostimulation exposures on sleep and recovery parameters in elite swimmers undergoing an intense training period. Twenty‐three elite French swimmers (7 females and 16 males) were involved in this controlled cross‐over protocol. The experiment took place during 2 weeks of intense training load. Each week (5 days and 5 nights) represented one of the two experimental conditions: partial body cryostimulation exposures (CRYO) or control sessions (CONT). Daily partial body cryostimulation exposure of 3 min at −110°C was performed (or not) during a consecutive period of 5 days, after the evening training session. Perceived wellness (anxiety, tiredness, depression and mood profile), sleep quality (via actimetry and cerebral recording) and nocturnal heart rate variability were evaluated. Collection of saliva samples permitted the measurement of C‐reactive protein and melatonin. Perceived anxiety, tiredness and depression were reduced after the CRYO week, concomitant with an improved mood profile. Recordings of cerebral activity during the night highlighted increased slow‐wave sleep duration in the first sleep cycle during the CRYO week. Other sleep parameters, including total sleep time, sleep latency, efficiency or movements during the night, remained unchanged. The concentration of C‐reactive protein in saliva was lower during the CRYO week compared with the CONT week. Moreover, sleep analysis allowed a distinction between better sleepers and poor sleepers. In the latter group, only poor sleepers among the male swimmers obtained a benefit on their sleep from cryotherapy. Repeated cryostimulation exposures during 1 week of intense training improved perceived wellness in elite swimmers, reduced inflammation, and modulated sleep architecture by increasing slow‐wave sleep duration.

## Abstract (highlights)

- **What is the central question of this study?**
  We hypothesized that chronic cryostimulation sessions during intensive training would have a positive impact on sleep quality, wellness feelings, inflammation and cardiac autonomic balance.
- **What is the main finding and its importance?**
  Repeated cryostimulation exposures during 1 week of intense training improved perceived wellness in elite swimmers, reduced inflammation and modulated sleep architecture by increasing slow‐wave sleep duration.

## 1 INTRODUCTION

High‐level athletes undergo periods of intensified training load many times in their preparation, primarily before the taper phase preceding competitions. Swimmers are particularly subject to these periods of increased load, with the objective of providing sufficient stimuli to trigger physiological adaptations. Intense training (IT) can lead to accumulation of fatigue, even more so when proper recovery is not achieved, possibly leading to reduced subsequent performance. To optimize recovery, a large variety of techniques are used by the elite athlete population: stretching, compression garments, massage, electrostimulation, contrasted water baths and cold therapies. This last category refers mainly to cold‐water immersion and cryostimulation. The technique of cryostimulation consists of a short exposure to very cold air (−110°C), including the head [whole‐body cryostimulation (WBC)] or not [partial‐body cryostimulation (PBC)], to induce physiological effects that are beneficial to athletes’ recovery. Among these effects, the most interesting are related to improved physical and psychological wellness, decreased inflammatory response (Pournot et al., 2011), activated parasympathetic cardiac tone and reduced muscle soreness (Bouzigon et al., 2021).

Moreover, cryostimulation might have beneficial effects on sleep (in terms of quality and quantity), which is one of the fundamental pillars of sport recovery and has been found to be greatly degraded in athletes, especially during IT periods (Gupta et al., 2017; Halson, 2019). Optimization of sleep quality and quantity is a challenge, and cryostimulation appears to be an interesting strategy through which it could be promoted. Until now, however, only a few studies have investigated its impact, even though it has been shown that after training, 3 min exposure in a PBC cabin enables improved subjective sleep quality and improved restless sleep (Bouzigon et al., 2014; Douzi et al., 2019b, 2019a). Nevertheless, few studies have tested the effect on sleep of several days of exposure to cryotherapy. In periods of IT, during which several consecutive high‐load days are completed, repeated exposures to cold might have a stronger impact. Indeed, the purpose of daily exposure during IT periods is to allow the athlete to endure high or higher training loads for several days, while limiting excessive fatigue and maintaining optimal sleep. A previous investigation tried to determine whether daily exposure to cryostimulation in elite athletes preserved sleep variables during a period of increased training stress (Schaal et al., 2015). It was shown that the signs of functional overreaching were lower, forestalling increased fatigue and the reduced quantity of sleep.

Regarding sleep analysis, numerous studies investigating sleep quality rely only on subjective indices and/or actimetry measurements. These parameters provide information such as time spent in bed, total sleep time and estimated awake stages. However, the results are not based on EEG measurements. In contrast, recently developed tools (e.g. the Dreem headband) now allow more accurate analysis of sleep structure via cerebral activity sensors, which are able to detect the different sleep stages in a less burdensome way than the gold‐standard polysomnography method (Arnal et al., 2020). The cerebral activity sensors used in this new apparatus can detect the successive 90 min sleep cycles occurring during a regular night of sleep. More precisely, they are able to distinguish the different stages in each cycle [i.e., the rapid eye movement (REM) sleep stage and the following three non‐REM sleep stages: Stage 1 or N1, the changeover from wakefulness to sleep; Stage 2 or N2, considered as a period of light sleep; and Stage 3 or N3, considered as a period of deeper sleep, also called slow‐wave sleep (SWS)]. Deep sleep is criucial to restorative functions, facilitating tissue recovery and strengthening the immune system, whereas REM sleep is believed to have a role with regard to cognitive functions, such as learning and creativity (Cai et al., 2009; Patel et al., 2021). For high‐level athletes, the SWS period is of major importance, especially insofar as growth hormone is produced mainly during this specific sleep stage (Leger et al., 2018), because its release stimulates muscle protein synthesis and, possibly, contributes to muscle recovery (Dijk, 2009; Halson et Julif, 2017).

The aim of the present study was to evaluate the effects of daily PBC exposures on sleep and recovery parameters in elite swimmers undergoing an IT period. We hypothesized that chronic PBC sessions following each of a consecutive 5 days of IT would have a positive impact on sleep quality, wellness feelings, inflammation and cardiac autonomic balance. We also postulate that this positive effect on sleep could depend on several characteristics of participants, such as sex and the profile of the sleeper. Of note, this study is one of the first to use a validated wireless headband enabling sleep monitoring and sleep stage detection in elite athletes.

## 2 MATERIALS AND METHODS

### 2.1 Protocol

#### 2.1.1 Ethical approval

The protocol was reviewed and approved by a French ethical committee for biomedical research (no. ID‐RCB: 2020‐A03563‐36), and all procedures were conducted in accordance with the principles of the *Declaration of Helsinki* (except for registration in a database).

#### 2.1.2 Participants

Twenty‐three elite swimmers participated in the study. Sixteen male swimmers (age, 19.1 ± 2.2 years old; body height, 1.83 ± 0.1 m; body mass, 73.1 ± 8.4 kg; best personal performance, 15.7% ± 5.2% of the world record) and seven female swimmers (age, 20.1 ± 2.9 years old; body height, 1.73 ± 0.1 m; body mass, 66.4 ± 8.7 kg; best personal performance, 14.5% ± 6.0% of the world record) without injuries or contraindication to cold exposure (Bouzigon et al., 2021) gave their written informed consent to participate in the study. All of them were considered Tier 3 swimmers according to the classification by McKay et al. (2022), i.e., highly trained/national level.

#### 2.1.3 Experimental design

**FIGURE 1:**
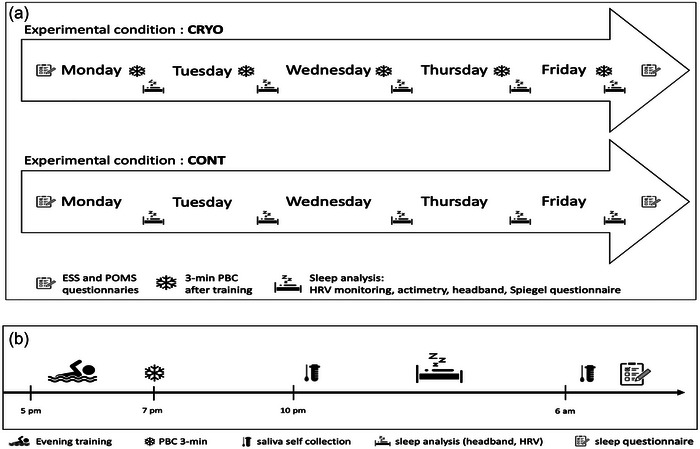
(a) General design of the study: two experimental conditions, CRYO and CONT. (b) Details of a specific day of the protocol (CRYO: cryotherapy, CONT: control, PBC: partial body cryotherapy, HRV: heart rate variability).

This study was designed as a randomized controlled cross‐over protocol. The experiment took place during a consecutive period of 2 weeks of intense training. Each week represented one of the two experimental conditions, with either PBC exposures (CRYO) or control sessions (CONT). Swimmers were assigned randomly to one of the two conditions for the first week and to the other condition in the second week of the protocol. As shown in Figure 1a, during five consecutive nights, from Monday to Friday, sleep was analysed in each condition. A PBC session was performed after each evening training session in the CRYO condition. No other recovery methods were implemented during the protocol, whatever the condition (i.e., CRYO or CONT). The organization of a typical day of the study is presented in Figure 1b.

#### 2.1.4 Partial body cryostimulation

Partial body cryostimulation sessions took place during a period of 5 days consecutively, from Monday to Friday, as soon as possible after completion of the evening training session (19.00 h). Each PBC session consisted of a 3 min exposure in a cryo‐cabin cooled to −110°C. Participants entered in the cryo‐cabin (head out) wearing gloves, slippers, underwear, a cap and a surgical mask. The cryo‐cabin was installed in a converted truck that was parked close to the pool to allow quick post‐workout exposure.

### 2.2 Measurements

#### 2.2.1 Perceptual measures

Before and after each experimental week, participants completed the Epworth Sleepiness Scale (ESS) and the Profile Of Mood States (POMS) questionnaire (McNair, 1971). After each experimental week, participants were asked to score from 0% to 100% the perception of their recovery.

#### 2.2.2 Sleep analysis

Sleep was monitored for five consecutive nights (from Monday to Friday) during the two experimental weeks through objective and subjective measurements. Sleep was assessed using objective measurements, such as movement and electrical brain activity, in addition to cardiac autonomic control and perceptual measures. During this experiment, bedtime and wake time were not imposed, and participants were instructed to keep their usual sleep schedules.

#### 2.2.3 Subjective measurements

Perceived sleep quality was evaluated using the six‐item Spiegel Questionnaire (Spiegel, 1984), and daytime sleepiness was assessed by the eight‐item Epworth Sleepiness Scale (Johns, 1991). Both questionnaires were administered during the morning after each experimental night.

#### 2.2.4 Objective measurements

Sleep quantity and architecture were assessed by means of a wireless‐connected headband (Dreem 2, Paris, France), validated for sleep‐related physiological signal recording and for sleep stage detection (Arnal et al., 2020). This electronic device is equipped with cerebral activity sensors that enable the estimation of several sleep parameters, including sleep onset latency, durations of sleep stages (N1, N2, N3 and REM), wake episodes, wake after sleep onset (WASO) and sleep efficiency. The quantity of movement during the night was assessed using an accelerometer worn on the non‐dominant wrist (WGT3X‐BT monitor, Actigraph, Pensacola, FL, USA). Both devices were used during each experimental night. This measurement scheme has already been used in several studies related to sleep analysis (Douzi et al., 2019a, 2019b, Aloulou et al., 2020, Arc‐Chagnaud et al., 2024a, 2020). We distinguished ‘better’ and ‘poor’ sleepers in our population of swimmers; they were categorized according to the time spent awake during the night (performed with a median split), which is relative to the duration of awakenings, an objective index of sleep quality.

#### 2.2.5 Movements

Actimetry was used to quantify movements during the nights. Subjects were instructed to wear an Actigraph (WGT3X‐BT monitor) on their non‐dominant wrist during each night of the protocol. Counts on the three‐dimensional axis were recorded during the night, and various sleep parameters were estimated: sleep efficiency, number of awakenings and their duration, and sleep fragmentation index (Aloulou et al., 2020; Douzi et al., 2019b; Arc‐Chagnaud et al., 2024a, 2020).

#### 2.2.6 Autonomic cardiac control

Participants wore a Polar H10 heart rate sensor connected to a Polar watch (Polar V800, Kempele, Finland) to measure R–R intervals during each experimental night. Three time windows were considered for analyses: the entire sleep period; a 4 h period of sleep starting 30 min after the reported bedtime (Dupuy et al., 2013; Hynynen et al., 2010; Myllymäki et al., 2011); and the first 10 min SWS period. Presence of an SWS episode was determined based on the procedure proposed by Brandenberger et al. (2005). R–R intervals were edited and inspected visually in order that ectopic beats could be replaced by interpolated data from adjacent normal‐to‐normal (N–N) intervals. The standard deviation of N–N intervals (SDNN) and the root mean square difference of successive N–N intervals (RMSSD) were calculated from the 512s segment retained for heart rate variability (HRV) analysis. The same segment was resampled at 2 Hz and was detrended for subsequent analyses in the frequency domain. As recommended by the Task Force (1996), spectral analysis was performed with a fast Fourier transform to quantify the power spectral density of the low‐frequency (LF; 0.04–0.15 Hz) and high‐frequency (HF; 0.16–0.40 Hz) bands. Additional calculations included LF + HF, LF and HF expressed in normalized units (i.e., as a percentage of LF + HF), and the LF/HF ratio. An analysis of the Poincaré plots was performed to estimate SD1 (standard deviation of Poincaré plot perpendicular to the line‐of‐identity) and SD2 (standard deviation of the Poincaré plot along the line‐of‐identity), the two parameters recommended by the Task Force (1996). All analyses were carried out with Kubios HRV software (v.3.4.3, Kubios Oy, Kuopio, Finland).

#### 2.2.7 Saliva specimens

Saliva specimens were self‐collected by the participants every night before bedtime (∼22.00 h) and every morning at wake‐up time (∼07.00 h) and immediately stored in freezer until assay. The ELISA kits used for the determination of melatonin, α‐amylase, C‐reactive protein (CRP) and cortisol were purchased from Salimetrics (State College, PA, USA). The specimens for measuring melatonin were collected before bedtime, whereas the specimens for measuring α‐amylase, CRP and cortisol were collected after the wake‐up time. All the specimens from one subject were analysed in the same series. The intra‐assay variations were 7.0% and 4.0% for cortisol concentrations of 0.06 and 2.07 µg/dL, respectively; 7.4% and 3.9% for melatonin concentrations of 6.1 and 31.7 pg/mL; 2.5% and 7.2% for α‐amylase concentrations of 17.7 and 474.6 U/mL; and 2.0% for CRP concentrations between 96 and 1197pg/mL.

### 2.3 Statistical analysis

Standard statistical methods were used to calculate the means and SD. Gaussian distribution was verified by a Shapiro–Wilk test. A two‐way factorial ANOVA (condition × time) with repeated measures on time was performed to test the null hypothesis that measures were not different between groups and periods. A specific three‐way ANOVA (condition × time × sleeper status) was performed to test the null hypothesis that measures were not different between groups, periods and sleeper status (i.e., better sleeper or poor sleeper). Compound symmetry, or sphericity, was checked by the Mauchley test. When the assumption of sphericity was not met, the significance of *F* ratios was adjusted according to the Greenhouse–Geisser procedure when the epsilon correction factor was <0.75 or according to the Huynh–Feldt procedure when the epsilon correction factor was >0.75, with the objective being to control for a type I error. Multiple comparisons were made using the Bonferroni *post hoc* test. Student's paired *t* test compared the mean values for the week. The magnitude of the difference was assessed by Hedges’ *g* when comparing dependent samples and by Cohen's *d* when comparing independent samples. The magnitude of the difference was considered small (0.2 < *g* or *d* < 0.5), moderate (0.5 < *g* or *d* < 0.8) or large (*g* or *d* > 0.8). The significance level was set at 5% for all analyses, which were performed with Statistica (StatSoft, Tulsa, OK, USA). Figures were created with Prism v.5 (GraphPad, Boston, MA, USA).

## 3 RESULTS

### 3.1 Training load

The training load calculated with Foster method (km 'kilometer'× RPE ‘perception of effort’) was 193 ± 161 and 229 ± 188, and the training load calculated with average weekly kilometres was 43.9 ± 18.6 and 46.8 ± 22.0 in CONT and CRYO conditions, respectively. Training load did not differ in both conditions (*p* = 0.158).

### 3.2 Sleep quality

**FIGURE 2:**
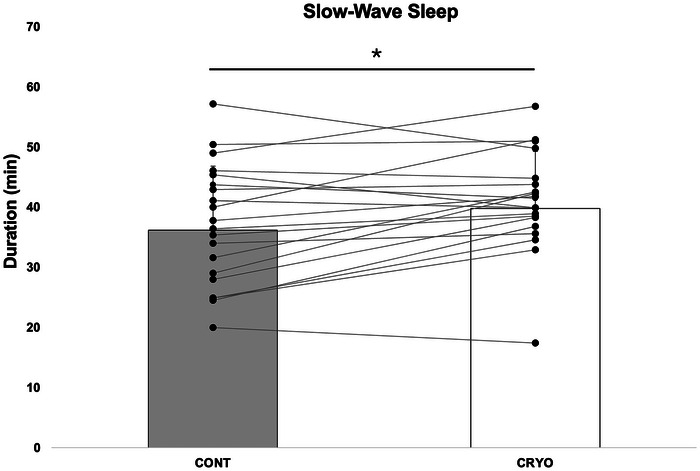
Slow‐wave sleep duration during the two experimental weeks (*n* = 23). ^*^Significant difference (*p *< 0.05) between CONT and CRYO conditions.

Sleep data collected through headband and actigraphy are presented in Table 1. The bedtime hours are 23.19 ± 00.53 and 23.14 ± 00.59 h for the control week and the cryostimulation week, respectively. The wake‐up times are at 07.08 ± 00.33 and 07.05 ± 00.47 h for the control week and the cryotherapy week, respectively. Sleep duration, sleep onset latency, sleep efficiency and the number and duration of awakenings did not change over the course of the week, whatever the condition. In contrast, Student's paired *t* test showed that the first SWS period of the night was longer in the CRYO condition than in the CON condition (*p* = 0,015 *g* = 0.34; Figure 2). Based on effect size, this effect seems more pronounced in swimmers considered as poor sleepers (*g* = 0.51) than in those considered as better sleepers (*g* = 0.12). Interestingly, we found an interaction between time × condition × sleep status and sex (*p* = 0.038), whereby we found increased SWS duration only on the fifth night and only among poor male sleepers (*p* = 0.034). The mean duration of SWS was 41.5 ± 16.4 min for male poor sleepers on the fifth night compared with the better sleepers, which was 26.0 ± 17.1 min.

**TABLE 1:** Sleep variables during the two experimental weeks (*n* = 23).

Assessment	Sleep indices		Night 1	Night 2	Night 3	Night 4	Night 5	Week
Questionnaire (Spiegel)	Sleep quality (score /30)	CONT	20.2 ± 3.6	19.8 ± 3.1	20.6 ± 2.5	20.3 ± 3.4	21.8 ± 2.6	20.5 ± 2.0
		CRYO	19.6 ± 3.3	19.4 ± 4.1	20.4 ± 3.3	21.3 ± 3.3	19.7 ± 5.6	20.1 ± 3.0
Accelerometer	Movements/TST	CONT	139 ± 56	141 ± 54	156 ± 72	141 ± 54	163 ± 67	148 ± 50
		CRYO	144 ± 69	145 ± 76	140 ± 63	138 ± 65	152 ± 54	144 ± 51
Headband	TST (min)	CONT	415 ± 55	417 ± 85	420 ± 71	407 ± 72	424 ± 62	416 ± 50
		CRYO	415 ± 67	415 ± 58	415 ± 59	415 ± 92	415 ± 79	417 ± 55
	SOL (min)	CONT	33.1 ± 22	27.2 ± 13.5	28.4 ± 18.6	28.2 ± 19.2	24.3 ± 16.4	28.2 ± 13.4
		CRYO	26.9 ± 18.5	32.9 ± 22.1	25 ± 13	23.4 ± 14.9	30.6 ± 19.4	27.7 ± 13
	Sleep efficiency (%)	CONT	89.8 ± 5.4	90.4 ± 6.2	90.3 ± 5.9	90.9 ± 5.9	91.5 ± 4.4	90.5 ± 3.9
		CRYO	90.2 ± 6.9	90.9 ± 5.6	91.3 ± 5.6	90.5 ± 6.7	89.5 ± 5.5	90.5 ± 4.6
	Wakes (a.u.)	CONT	10.1 ± 6.8	10.9 ± 7.9	10.7 ± 8.9	10.7 ± 9.0	11.0 ± 8.2	10.7 ± 6.1
		CRYO	10.8 ± 9.5	10.3 ± 6.3	10.1 ± 9.2	10.0 ± 10.0	10.1 ± 6.7	10.3 ± 6.8
	WASO (%)	CONT	3.3 ± 3.1	2.6 ± 2.4	3.2 ± 3.5	3.0 ± 3.5	4.7 ± 8.1	3.3 ± 3.0
		CRYO	4.3 ± 6.8	2.4 ± 1.9	2.7 ± 3.6	3.8 ± 7.4	3.1 ± 2.9	3.2 ± 3.9
	REM (%)	CONT	25.0 ± 7.8	25.3 ± 4.8	27.2 ± 5.1	26.5 ± 7.2	26.6 ± 7.1	26.1 ± 3.9
		CRYO	26.0 ± 7.6	27.0 ± 5.7	26.8 ± 5.2	25.1 ± 5.5	27.9 ± 5.7	26.5 ± 3.8

*Note*: Data are presented as the mean ± SD. Significant difference between CONT and CRYO conditions, *p *< 0.05.

Abbreviations: REM, rapid eye movement; SOL, sleep onset latency; TST, total sleep time; Wakes, number of awakenings relativized by sleep duration; WASO, wake after sleep onset.

### 3.3 Saliva analyses

**FIGURE 3:**
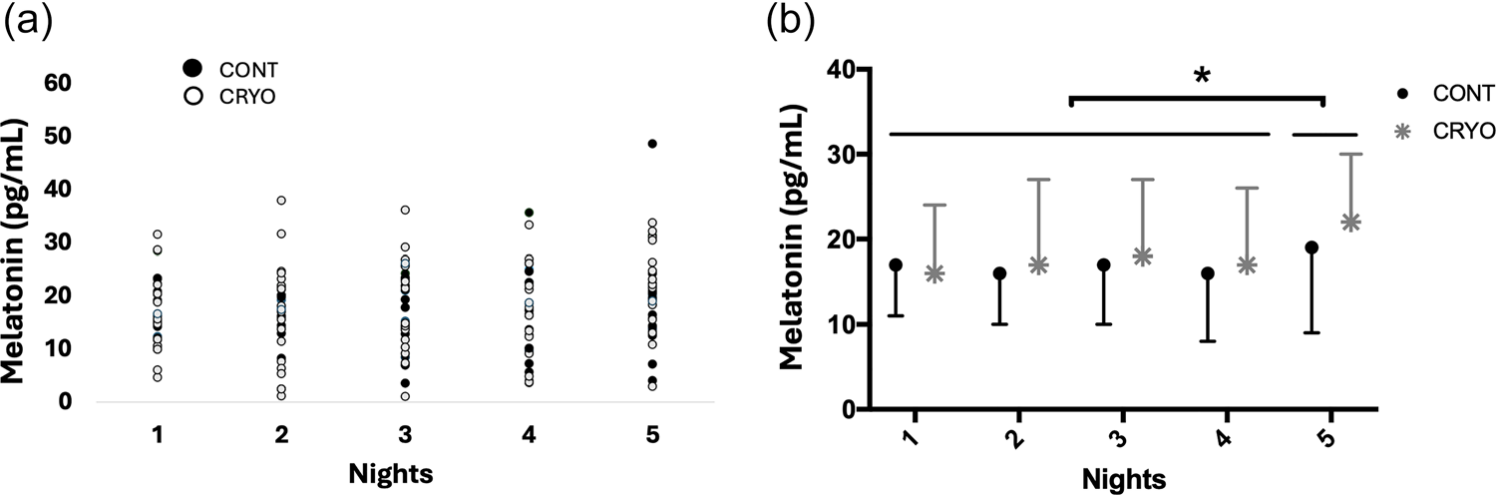
(a) Melatonin concentrations of individuals. (b) Mean melatonin concentrations ^*^Significant difference (*p *< 0.05) between Night 5 and the other nights.

**FIGURE 4:**
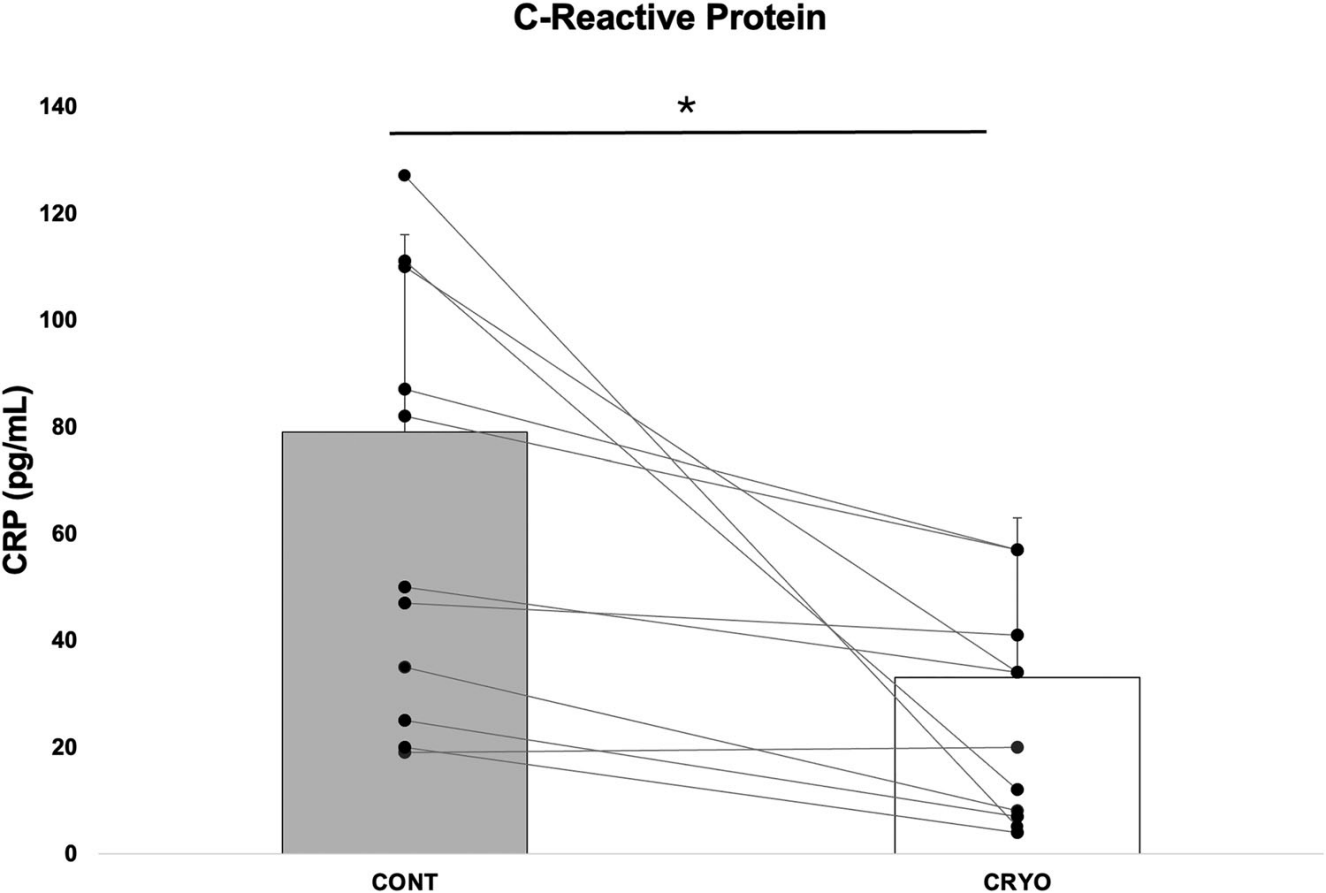
C‐reactive protein (CRP) concentrations. ^*^Significant difference (*p *< 0.05) between CONT and CRYO conditions (*n* = 13).

We found a tendency effect of time (*p* = 0.065) on melatonin, with the concentration before the fifth night being higher than the others (*p* = 0.01, *g* = 1.64), but without an interaction between time and group (Figure 3). No significant changes were observed in cortisol and α‐amylase concentrations. In contrast, we found an effect of condition on CRP (*n* = 13), which was systematically lower in the CRYO condition than in the CON condition (*p* = 0.007, *g* = −0.63; Figure 4.

### 3.4 Autonomic cardiac control

Regarding HRV parameters, we did not find any significant effect of time or condition, and no interaction. All the results are presented in Table 2.

**TABLE 2:** Heart rate variability parameters during the two experimental weeks (*n* = 23).

Heart rate variability parameters		Night 1	Night 2	Night 3	Night 4	Night 5	Week
HR (ms)	CONT	55.8 ± 7.0	55.4 ± 8.5	54.5 ± 7.7	54.2 ± 6.7	55 ± 5.6	55 ± 6.9
	CRYO	56.2 ± 6.2	54.2 ± 8.8	54.8 ± 7.1	55.1 ± 8.5	55.6 ± 9.1	55.1 ± 7.9
RR (ms)	CONT	1093 ± 147	1 106 ± 169	1124 ± 165	1124 ± 143	1100 ± 121	1109 ± 145
	CRYO	1083 ± 125	1138 ± 184	1107 ± 150	1114 ± 165	1106 ± 181	1110 ± 164
RMSSD	CONT	83.2 ± 33.1	81.5 ± 34.7	83.0 ± 40.5	84.5 ± 36.4	89.0 ± 34.3	84.3 ± 37.2
	CRYO	82.4 ± 39.3	87.4 ± 37.8	86.2 ± 39.6	88.4 ± 39.5	94.0 ± 40.6	87.8 ± 36.2
LF/HF	CONT	1.5 ± 0.7	1.7 ± 1.0	1.7 ± 1.1	1.7 ± 1.2	1.4 ± 0.9	1.6 ± 0.9
	CRYO	1.3 ± 0.8	1.5 ± 1.0	1.7 ± 1.2	1.8 ± 1.4	1.6 ± 1.2	1.6 ± 1
HFnu	CONT	44.4 ± 13.7	42.5 ± 15.0	42.4 ± 16.3	42.6 ± 15.9	46.6 ± 14.9	43.7 ± 14.8
	CRYO	48.8 ± 15.5	45.5 ± 16.2	44.2 ± 17.8	43.7 ± 17.0	45.4 ± 16.2	45.5 ± 15.3
LFnu	CONT	55.6 ± 13.7	57.5 ± 15.0	57.6 ± 16.3	57.3 ± 15.9	53.4 ± 14.9	56.2 ± 14.8
	CRYO	51.2 ± 15.5	54.5 ± 16.2	55.7 ± 17.8	56.3 ± 17.0	54.6 ± 16.2	54.5 ± 15.3

*Note*: Data are presented as the mean ± SD; Abbreviations: HFnu, normalized high‐frequency power; HR, heart rate; LF/HF, sympathovagal balance; LFnu, normalized low‐frequency power; RMSSD, mean root square of successive differences; RR, interval beat‐to‐beat.

### 3.5 Perceptual measures

**FIGURE 5:**
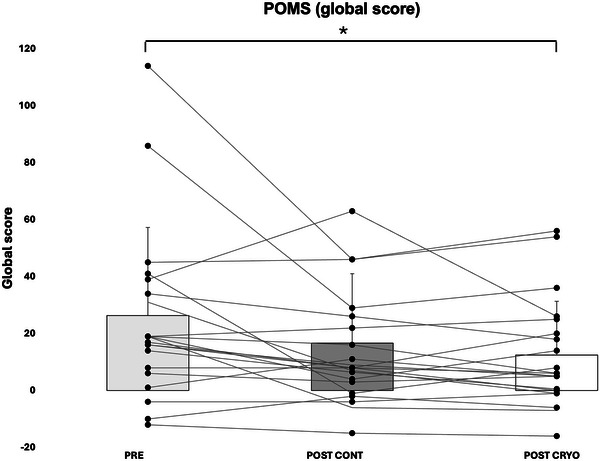
Global scores of the Profile Of Mood States (POMS). ^*^Significant difference (*p *< 0.05) between PRE and POST, for CRYO conditions.

The global score for the POMS was lower after the CRYO condition (Figure 5), indicating an improved mood state (*p* = 0.003). Anxiety, tiredness and depression were reduced, and shape index was enhanced after the CRYO condition compared with CONT (*p* = 0.004, *p* = 0.002, *p* = 0.003 and *p* = 0.02, respectively). None of the experimental conditions had an impact on perceived sleepiness of the participants, which was evaluated by the ESS after each week. Moreover, perception of recovery was greater after the week of daily PBC exposure than after the CONT week (59% vs. 41%, *p *< 0.05; 100% meaning a sensation of complete recovery).

## 4 DISCUSSION

This study is the first to evaluate the effects of daily cryostimulation exposure on sleep quality, nocturnal cardiac autonomic activity and inflammatory markers in elite swimmers during an IT period. Our results suggest that repeated PBC after training for a consecutive period of 5 days has a positive impact on perceived fatigue, recovery, mood and, depending on the sleep quality of each participant, possibly on systemic inflammation and sleep architecture.

Regarding subjective measures, swimmers had a better perceived recovery feeling after the CRYO week. They felt less anxious, tired and depressed, and felt in better shape and in a better state of mind after the CRYO session compared with the CONT one. This sense of improved wellbeing after cryostimulation has been observed after a single exposure (Bouzigon et al., 2021) and might be linked to modulation of the inflammatory response (Lombardi et al., 2017). In the present work, we showed that this feature is maintained during a week of IT followed every day by cryostimulation. In a cohort of healthy males having undergone repeated WBC sessions, Lubkowska et al. (2011) reported increased anti‐inflammatory cytokines concomitant with decreased pro‐inflammatory interleukin‐1α. When performed after training, WBC induced a reduced pro‐inflammatory response characterized by attenuated CRP levels until 3 days after exposure (Pournot et al., 2011). This component was also measured in our study, and CRP concentrations were lower during the 5 days of PBC exposure compared with the CONT condition. This result is similar to that reported by Ziemann et al. (2012), who observed reduced tumour necrosis factor‐α levels in tennis players who had undergone 5 days of WBC during a training camp.

Regarding sleep analysis, subjective sleep quality was similar in both weeks, with no apparent effect of PBC exposure. Cerebral activity recordings during the nights revealed similar objective sleep outcomes in both conditions: sleep duration, onset latency, number and duration of awakenings, and sleep efficiency did not change between CRYO and CONT sessions. However, daily PBC modified the sleep structure, inducing a longer duration of the first SWS period of the night. Also called ‘deep sleep’, SWS is of major importance for physical recovery. This sleep stage is characterized by the release of growth hormone, which stimulates protein synthesis and, consequently, favours muscle fibre repair (Dijk, 2009; Léger et al., 2018). Increased SWS duration is an underlying mechanism through which recovery can be optimized during IT days. In fact, these periods of stressful training are characterized by sleep disturbances (Fietze et al., 2009; Hausswirth et al., 2013; Taylor et al., 1997), and our study showed that repeated cryostimulation appeared to be an effective means of enhancing its quality. In a similar context involving synchronized swimmers, Schaal et al. (2015) demonstrated the usefulness of daily WBC as a means of preserving sleep duration and efficiency during a period of intensified training. That said, sleep was analysed by actigraphy, and specific sleep architecture could not be examined. This is indicative of the main limitation of studies investigating sleep quality. In most cases, sleep quality is determined by wrist actimetry and sleep diaries only, impeding the detection of sleep stages, which is directly feasible thanks to the recording of cerebral activity. On the contrary, with a focus on subjective ratings of sleep quality and actigraphy measurement, our participants did not exhibit benefits of daily PBC sessions, an outcome similar to that observed by Broatch et al. (2019), in whose study repeated WBC after training did not seem to impact sleep quality. These findings underscore the importance of accurate tools to evaluate variables such as sleep stages. Using polysomnography, Chauvineau et al. (2021) reported findings comparable to ours. The proportion of SWS was enhanced during the first part of the night, when intense exercise was followed by whole‐body cold‐water immersion. Although the cooling strategies differ, these results support the hypothesis that cold exposure can regulate sleep architecture. Recently, Arc‐Chagneaud et al. (2024a) found the same results in the general population, because 5 days of cryostimulation improved the SWS stage of sleep. In our study, this effect was particularly pronounced in poor male sleepers, once again raising the question of the differences between men and women in response to cryostimulation (Theurot et al., 2021, 2023).

Intense training is characterized by increased sympathoadrenal activation, which can affect the quality of passive recovery by impairing sleep (Driver et al., 1994; Netzer et al., 2001). Previous studies investigating the effects of IT periods revealed consequential HRV disruptions at rest, which were associated with increased fatigue and poor sleep quality (Al Haddad et al., 2009; Garet et al., 2004; Hynynen et al., 2006, 2010; Uusitalo et al., 2000) In this context, post‐training cooling strategies might reduce the usual exercise‐induced decrease in parasympathetic activity. In a previous study, better HRV was observed in the morning and associated with an improved rating of perceived sleep quality, using, daily for 5 days, a 5 min cold‐water immersion post‐training (Al Haddad et al., 2012). In fact, following cold exposure, the autonomic nervous system is characterized by predominance of the parasympathetic drive, which is usually associated with faster postexercise recovery (Louis et al., 2015, 2020). This feature has been observed previously in cases where HRV was recorded immediately after cryostimulation (Theurot et al., 2021). However, we did not observe any such overall feature in our study. This non‐detection of a greater parasympathetic tone might have arisen from a nocturnal HRV analysis that began a few hours after PBC exposure, and not immediately after. All in all, we did not observe any effect of cryostimulation on HRV‐related indices during the five consecutive nights.

Several physiological mechanisms might explain how cryotherapy could improve sleep. One primary mechanism is the reduction in core body temperature after cold exposure. Cryotherapy is known to decrease internal temperature (Arc‐Chagneaud et al., 2024b), a process often associated with better sleep quality. This temperature drop signals the body to prepare for sleep, facilitating faster sleep onset (Herberger et al., 2024). Additionally, the cooling effect might promote deeper sleep and enhance the proportion of SWS sleep (Herberger et al., 2024). Another potential explanation, although not directly addressed in this article, is the activation of the parasympathetic nervous system observed after cold exposure. The parasympathetic nervous system is associated with relaxation and stress reduction, both of which are associated with better sleep. Cold exposure might shift the autonomic balance towards parasympathetic dominance, potentially leading to more restorative sleep, particularly during SWS stages (Arc‐Chagneaud et al., 2024b; Douzi et al., 2019b). Finally, the anti‐inflammatory properties of cryotherapy might also contribute to improved sleep quality. Chronic inflammation is a known disruptor of sleep patterns (Besedovsky et al., 2019), and cold exposure might help to mitigate this by reducing pro‐inflammatory cytokines (Pournot et al., 2011). This effect could be particularly beneficial for individuals suffering from sleep disturbances linked to inflammation‐related conditions.

Further research is required to identify the strategy most likely to enhance the effect of sleep quality. Cryostimulation appears to be an effective tool owing to its multiple beneficial effects on recovery parameters. However, more studies are needed to determine the ideal timing of exposure after training, with the objective being to maximize potential positive effects on subsequent sleep. Also, future research on cryotherapy and sleep improvement should focus not only on sex‐specific effects but also on individual sleep profiles. Our study suggests that the effects of cryotherapy might vary depending on the profile of the sleeper, with poorer sleepers appearing to benefit more from this intervention. Understanding these differential responses could help to optimize cryotherapy protocols for sleep enhancement.

## 5 CONCLUSION

Repeated exposure to cryostimulation during IT significantly enhances sensations of wellbeing and overall recovery in competitive swimmers by modulating sleep architecture and inflammation.

## AUTHOR CONTRIBUTIONS

Coralie Arc‐Chagneaud: conception and design of the work, acquisition, analysis and interpretation of data for the work, drafting the manuscript and revising it critically for important intellectual content. Benoit Dugué and Olivier Dupuy: conception and design of the work, analysis and interpretation of data for the work, drafting the manuscript and revising it critically for important intellectual content. Robin Pla, Romain Bouziguon and Laurent Bosquet: and interpretation of data for the work, drafting the manuscript and revising it critically for important intellectual content. All authors approved the final version of the manuscript and agree to be accountable for all aspects of the work in ensuring that questions related to the accuracy or integrity of any part of the work are appropriately investigated and resolved. All persons designated as authors qualify for authorship, and all those who qualify for authorship are listed.

## CONFLICT OF INTEREST

None declared.

## Data Availability

The data are available on reasonable request.
